# TVA–based assessment of attentional capacities–associations with age and indices of brain white matter microstructure

**DOI:** 10.3389/fpsyg.2014.01177

**Published:** 2014-10-21

**Authors:** Thomas Espeseth, Signe A. Vangkilde, Anders Petersen, Mads Dyrholm, Lars T. Westlye

**Affiliations:** ^1^Department of Psychology, University of OsloOslo, Norway; ^2^Norwegian Centre for Mental Disorders Research (NORMENT), and KG Jebsen Centre for Psychosis Research, Division of Mental Health and Addiction, Oslo University HospitalOslo, Norway; ^3^Department of Psychology, Center for Visual Cognition, University of CopenhagenCopenhagen, Denmark

**Keywords:** cognition, processing speed, fluid intelligence, diffusion tensor imaging, fractional anisotropy, diffusivity

## Abstract

In this study the primary aims were to characterize the effects of age on basic components of visual attention derived from assessments based on a theory of visual attention (TVA) in 325 healthy volunteers covering the adult lifespan (19–81 years). Furthermore, we aimed to investigate how age-related differences on TVA parameters are associated with white matter (WM) microstructure as indexed by diffusion tensor imaging (DTI). Finally, we explored how TVA parameter estimates were associated with complex, or multicomponent indices of processing speed (Digit-symbol substitution, *DSS*) and fluid intelligence (*g*F). The results indicated that the TVA parameters for visual short-term memory capacity, *K*, and for attentional selectivity, α, were most strongly associated with age before the age of 50. However, in this age range, it was the parameter for processing speed, *C*, that was most clearly associated with DTI indices, in this case fractional anisotropy (FA), particularly in the genu and body of the corpus callosum. Furthermore, differences in the *C* parameter partially mediated differences in *DSS* within this age range. After the age of 50, the TVA parameter for the perceptual threshold, *t*_0_, as well as *K*, were most strongly related to participant age. Both parameters, but *t*_0_ more strongly so than *K*, were associated WM diffusivity, particularly in projection fibers such as the internal capsule, the sagittal stratum, and the corona radiata. Within this age range, *t*_0_ partially mediated age-related differences in *g*F. The results are consistent with, and provide novel empirical support for the neuroanatomical localization of TVA computations as outlined in the neuronal interpretation of TVA (NTVA). Furthermore, the results indicate that to understand the biological sources of age-related changes in processing speed and fluid cognition, it may be useful to employ methods that allow for computational fractionation of these multicomponent measures.

## Introduction

Cognitive abilities, such as perception, attention, memory, and language depend on the precise temporal coordination of a spatially distributed set of neural computations that are organized in brain networks of various sizes and complexities (McClelland et al., 1986; Mesulam, 1990). Efficient communication between distal brain regions is thought to rely on the integrity of the white matter (WM) tracts that connect them (Neubauer and Fink, 2009). Neurological disease that involves disruptions in the structural connectivity within and between brain networks leads to cognitive dysfunctions (Geschwind, 1965a,b; Catani and Ffytche, 2005), suggesting that the integrity of WM tracts may provide a neuroanatomical substrate for individual differences in cognitive abilities (Bressler and Menon, 2010). Consistent with this, variation in network structural connectivity has been linked to cognitive function in younger adults (Gold et al., 2007; Turken et al., 2008) and in age-heterogeneous samples (Kennedy and Raz, 2009; Kochunov et al., 2010; Penke et al., 2010; Madden et al., 2012; Salami et al., 2012). In normal aging, general fluid intelligence (*g*F) declines earlier and more rapidly than general crystallized intelligence (*g*C) (Salthouse, 2004; Craik and Bialystok, 2006). While *g*C is defined by tasks that measure the ability to apply acquired knowledge and learned skills, *g*F refers to the ability to reason in novel situations (Cattell, 1963; Carroll, 1993). Processing speed is a well-known and highly replicated correlate of *g*F that has been hypothesized to mediate the relation between the brain and *g*F (Jensen, 2006) and also to drive age-related cognitive decline (Salthouse, 1996). However, processing speed itself is a complex phenomenon, including perceptual, attentional, memory, and motor components, each of which may have differential relations with WM networks and their biological properties.

Diffusion tensor imaging (DTI) is a powerful tool for quantifying structural integrity of brain networks. DTI is sensitive to the direction and degree of water displacement in biological tissues (Beaulieu, 2002). Diffusion in brain parenchyma is restricted by cytoskeletal axonal elements including plasma membranes, microtubules and myelin sheaths (Beaulieu, 2002). Water displacement occurs faster along than across the axons, and DTI enables visualization and quantification of the local organization of WM pathways via measures of directionality and rate of molecular diffusion as a function of a tensor ellipsoid that is estimated for each voxel. The most commonly reported measure is fractional anisotropy (FA), which indexes the fraction of the restricted (or anisotropic) diffusion relative to the total diffusion within each voxel. The FA range is 0–1 and values closer to 1 indicate increased directionality of diffusion. The average rate of diffusion along all directions is indexed by mean diffusivity (MD), which is orthogonal to FA. MD can be further split into rate of diffusion along the primary (axial diffusivity, AD) and the secondary (radial diffusivity, RD) axes of the diffusion ellipsoid. Whereas the exact neurobiological correlates of the DTI indices are unknown and likely to be complex, animal studies have suggested that AD may be sensitive to axonal changes, whereas RD may be sensitive to myelin changes (Song et al., 2003, 2005; Sun et al., 2006, 2008). Studies investigating effects of age on DTI measures in cross-sectional designs have shown that after the age of about 40 years, FA is negatively correlated with age, whereas MD is positively correlated with age (Westlye et al., 2010), and this general trend has been confirmed in longitudinal studies (Barrick et al., 2010).

Information processing speed is a fundamental capacity across the adult life span that correlate with the performance on many different kinds of cognitive tasks (Kail and Salthouse, 1994; Li et al., 2004). Higher *g*F has been shown to be associated with higher FA and lower MD, AD, and RD, and most of this association seems to be mediated by processing speed (Penke et al., 2012a; Haász et al., 2013). However, processing speed as typically measured in behavioral tasks is in itself a complex variable, the components of which may be mediated by distinct brain networks. For example, there is usually a motor component in estimates of processing speed which, when statistically controlled for, removes or significantly alters the pattern of association with DTI indices (Bennett et al., 2012). The influence of the motor component can be alleviated through mathematical modeling of the reaction time distribution (Ratcliff, 1978), or by the use of accuracy data from tasks with briefly presented stimuli, such as the Useful Field Of View (UFOV) task (Ball and Owsley, 1993), or the Inspection Time (IT) task (Deary and Stough, 1996). However, with briefly presented stimuli, a failure to accurately identify an object may be attributed either to an elevated threshold of perception, or to slower recognition processes, or both. The UFOV and the IT do not differentiate between these two factors.

Bundesen's (Bundesen, 1990) Theory of Visual Attention (TVA) offers an approach to measuring processing speed that is independent of motor speed while dissociating perceptual threshold and processing speed. TVA can be seen as a mathematical formalization of the influential biased competition model of attention (Desimone and Duncan, 1995), according to which objects compete for cortical representation and limited processing resources, and the competition is biased by bottom-up and top-down factors. According to TVA, selection and recognition of stimuli in the visual field occurs simultaneously during a process termed *visual categorization* (Bundesen, 1990). A visual categorization entails a decision as to whether “object *x* has feature *i*,” or equivalently “object *x* belongs to category *i*.” When such a categorization is made, object *x* is recognized as member of category *i*, and is thereby selected. However, for the categorization to be completed, it needs to be encoded into a visual short-term memory (VSTM) store, which is of limited capacity. In TVA, visual attention is described as a parallel processing race in which stimuli compete for representation in VSTM. The competition is influenced by attentional weights and perceptual biases, with the effect that the probability of representation in VSTM is biased in favor of particular stimuli in the visual field. The allocation of weights and biases are specified in the *rate equation* and the *weight equation* of TVA. According to the *rate equation:*

$$v\left(x,i\right)=\;\eta (x,i)\beta_{i}\frac{w_{x}}{\sum_{z\;\in \;S}w_{z}},$$

the rate of processing *v*(*x,i*) (i.e., of the categorization “object *x* has feature *i*”) is given by the product of, η(*x,i*), the strength of the sensory evidence that object *x* belongs to category *i*, β_*i*_, the perceptual bias associated with *i*, and the attentional weight of object *x*, *w*_*x*_, divided by the summed attentional weights, *w_z_*, of the set of elements in the visual field, *S*. The attentional weights are given by the *weight equation*:

$$w_{x}=\;\sum_{j\;\in \;R}\eta (x,j)\pi_{j},$$

which specifies that the attentional weight of object *x*, *w_x_*, is given by the product of the sensory evidence that “object *x* belongs to category *j*,” η(*x,j*), and the pertinence value associated with category *j*, π_*j*_, across the set of perceptual categories, *R*. The pertinence of category *j* is a measure of the momentary importance of attending to objects that belong to category *j* (e.g., the importance of the category red when looking for red objects) (Bundesen and Habekost, 2008). Thus, the attentional weight of object *x* is the sum of all pertinence values, weighted by the degree of sensory evidence that object *x* is a member of category *j*.

In NTVA, Bundesen et al. (2005) offers a neurophysiological interpretation of TVA in which the total neural activation representing a visual categorization is proportional to the *number* of neurons representing the categorization, and the *activation level* of these. The number of neurons representing the categorization of object *x* is given by *w_x_*/Σ*w_z_*, whereas the activation level of these neurons is directly proportional to β_*i*_. The implementation of visual categorizations is characterized by two processing waves. Visual processing resources (i.e., neurons) are first distributed at random among objects in the visual field, and a parallel matching process between objects *x* in neuronal receptive fields and long-term memory representations *i*, resulting in sensory evidence values, η(*x,j*), unfolds. Building on this evidence, attentional weights are computed for each object in the visual field, and are used for redistribution of cortical processing capacity across them. In particular, a priority map, representing the attentional weights, controls dynamic remapping of neuronal receptive fields to ensure that the number of cells allocated to a particular object becomes proportional to the attentional weight of the object. Subsequently, different categorizations compete for access to VSTM in a stochastic race process. The capacity of VSTM is limited to *K* elements, typically around 4. The first *K* visual objects to finish processing are stored in VSTM, are accessible to consciousness and can be reported. The remaining categorizations are lost.

TVA-based assessment is typically done by use of simple letter identification tasks such as *whole report* (Sperling, 1960), in which an array of letters is briefly presented and participants are asked to report back the identity of as many letters as they can, or *partial report* (Shibuya and Bundesen, 1988), where participants should report back the identity of only a subset of the letters, for example only those printed in a certain color. The dependent measure in these tasks is accuracy (i.e., number of correctly identified letters). When the rate and weight equations are fitted to accuracy data from a combined whole report and partial report task, values on five distinct mathematical parameters can be estimated for each individual participant (Duncan et al., 1999). TVA defines these as the storage capacity of visual short-term memory, *K*; the perception threshold, *t*_0_; visual processing speed, *C*; visual distractibility or selectivity, α; and the relative attentional weight of each visual object, *w*. The *w* parameter can be used to compute the relative balance between attentional weights in the left and right visual fields, *w*_index_. Parameter estimates are strongly dependent on task characteristics (e.g., stimulus types and contrasts), but has proved to be valid and reliable measures, with split-half reliabilities around 90% for all parameters, and test-retest reliabilities ranging from around 60% (*t*_0_, *C*, α), to around 90% (*K* and *w*_index_) (Habekost et al., 2014).

Two studies on effects of age on TVA parameters have been published to date (McAvinue et al., 2012; Habekost et al., 2013). McAvinue and colleagues used the *CombiTVA* task (Vangkilde et al., 2011) and studied 113 individuals aged 12–75 years (83 participants in the 20–75 age range). They reported linear functional decline with age for the parameters *K, t*_0_, *C*, and α, with the steepest slopes for *C* and *K*, and weaker, but significant effects for *t*_0_ and α. Although the regression analyses indicated that all age effects were linear, closer inspection of the local weighted scatterplot smoothing (LOESS, Cleveland, 1979) fits showed that *t*_0_ and α might have more complex relationships with age; *t*_0_ appeared to be relatively stable from the teens to the late 50s, but increase thereafter (from about 17 to about 23 ms). In contrast, α increased already from the teens and until the age of about 50, but appeared to be relatively stable thereafter. Habekost et al., (2013) studied an older age cohort (*n* = 33, age range 69–87) in two experiments. The first experiment aimed to disentangle perceptual speed from perceptual threshold and entailed a whole report procedure in which single letters were presented at fixation at different exposure times. The demands in this task are somewhat different from those of the *CombiTVA* and the nominal value of the parameter estimates cannot be directly compared to those of McAvinue and colleagues. However, this study also reported particularly strong age trends for processing speed, *C* (~50% decrease, *R*^2^ ~23%), as compared to the perceptual threshold, *t*_0_(*R*^2^ ~9%). Interestingly, they also reported a negative correlation between *C* and the Fazekas rating of white matter hyperintensities (Fazekas et al., 1987), but this correlation did not withstand controlling for participant age. The *t*_0_ results showed that there was a small group of the oldest participants that had elevated parameter estimates and the authors interpreted this finding to indicate potential pathological decline in this subgroup, in line with previous research reporting that individuals with mild cognitive impairment have significantly higher perceptual thresholds than age-matched healthy controls (Bublak et al., 2011). The second experiment assessed the participant's visual span with a whole report task in which five letters were presented peripherally for a fixed exposure time of 200 ms. Visual span was negatively correlated with age, but the association was weaker than for processing speed (*R*^2^ ~9%).

Summarized, fluid intelligence declines rapidly in normal aging, and it is believed that this is partly driven by changes in brain structural connectivity. Furthermore, the association between connectivity and fluid intelligence may be mediated by changes in processing speed. However, processing speed consists of several subcomponents, each of which may be subserved by distinct brain networks and biological mechanisms. Thus, in the present study we aim to investigate these issues by (1) describing the effects of age on specific components of processing capacities as defined by TVA and on computationally more complex measures of psychometric processing speed and fluid intelligence, (2) analyzing the effects of age on different DTI indices of WM tract integrity, (3) exploring the associations between the behavioral measures and DTI indices, including an analysis of potential meditational effects of DTI indices on age-related TVA parameter differences, and of potential meditational effects of TVA parameters on the relation between DTI indices and *DSS* and *g*F. Finally, (4) we explore the regional specificity of these brain-behavior correlations.

## Materials and methods

### Sample recruitment and demographics

Participants were drawn from the Norwegian Cognitive NeuroGenetics (NCNG) sample which were recruited by advertisements in a local newspaper to take part in a larger community based study on the genetics of cognition (see Espeseth et al., 2012) for an overview). All participants read an information sheet and signed a statement of informed consent approved by the Regional Committee for Medical and Health Research Ethics (South-East Norway) (Project ID: S-03116). Permission to obtain and store blood samples for genotyping, as well as cognitive and MRI data in a biobank, and to establish a registry with relevant information, was given by the Norwegian Department of Health. The research was carried out in compliance with the Helsinki Declaration. All participants were native speakers of Norwegian. All subjects were interviewed and screened for neurological or psychiatric diseases known to affect the central nervous system, and history of substance abuse. Any person with a history of treatment for any of the above was excluded from further participation. The participants were administered the Vocabulary and Matrix reasoning (MR) subscales of the Wechsler Abbreviated Scale of Intelligence (*WASI*, Wechsler, 2007) to estimate general cognitive abilities (IQ). The behavioral sample consisted of three hundred and twenty five persons (219 females) in the age range 19–81 (Mean = 50.2, *SD* = 17.0). Participants included in the study had on average 14.6 years of education (*SD* = 2.3, range = 9–22) and performed within an estimated full scale IQ range of 88–148 (Mean = 121, *SD* = 10.2). Mini-Mental State Examination (MMSE) data was available for 209 individuals aged > 40 (Mean = 29.1, *SD* = 0.9, range = 26–30). Concurrent TVA and DTI data was available for two hundred and twenty nine participants. This subsample consisted of 149 females. Demographics values were similar to those from the total sample. All images were checked for signs of pathology by a certified neuroradiologist.

### Behavioral tasks

#### Neuropsychological test battery and estimation of gF

All participants completed a battery of neuropsychological tests in addition to the *WASI*, all tests in the official Norwegian language version. This battery included measures of episodic memory (California Verbal Learning Test, second version, *CVLT-II*) (Delis et al., 2004) and measures of processing speed. Processing speed was derived from performance on the Color–Word Interference Test (*CWIT*) (Delis et al., 2005), from an experimental visuo-spatial attention task involving letter discrimination with location cues of varying validity (*CDT*) (Espeseth et al., 2006), and from the Digit-Symbol Substitution (*DSS*) test (Wechsler, 1981). To construct *g*F factor scores, hierarchical PCA analyses were performed (see Davies et al., 2011). Data from *WASI MR, CVLT-II, CWIT*, and *CDT* was used as input to the PCA. Raw scores from *CWIT* and *CDT* were inverted to obtain the same ordinal order as the two other test scores (i.e., higher score = better function). Initially, two separate first-order PCAs were run on *CVLT-II* and inverted *CWIT* scores. Thereafter, a second-order PCA was run on the first, un-rotated component factor scores obtained from *CVLT-II* and *CWIT* subtests, raw MR scores and the inverted *CDT* scores. From this, the first un-rotated principal component was extracted and used to represent *g*F. Component scores such as *g*F are known to be highly reliable, even when derived from different batteries of cognitive tests (Johnson et al., 2004, 2008). Although no direct comparison between different *g*F estimations, or test-retest reliability estimates, have been conducted in the current sample, *g*F association with age, genetics, and DTI indices have been found to be comparable to such associations in other samples (Davies et al., 2011; Haász et al., 2013; Christoforou et al., 2014).

#### TVA-bases assessment—design and procedure

**Figure 1:**
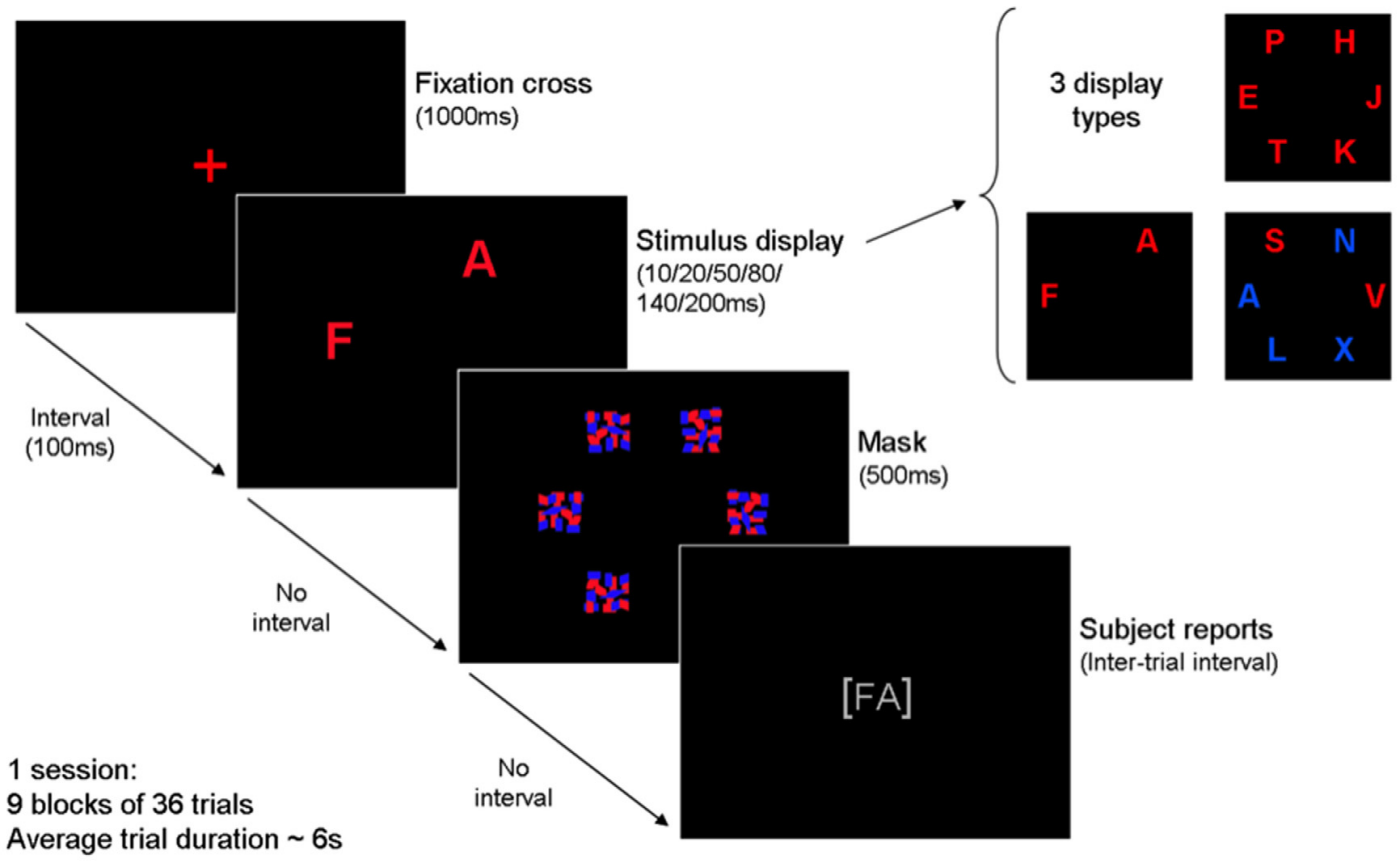
**Outline of a single trial in the *CombiTVA* paradigm showing timing and three types of letter displays used: six target whole report (red letters), two target whole report (red letters), and two target and four distractor partial report (red and blue letters)**.

The *CombiTVA* paradigm, which was employed in the present study, is described in detail in Vangkilde et al. (2011). Briefly, the *CombiTVA* test took 45 min to complete and comprised 24 practice trials and nine experimental blocks of 36 trials. All trials followed the same basic design outlined in Figure 1.

A trial was initiated by a red fixation cross in the middle of a black screen, and then a 100-ms blank screen, before the stimulus display was presented on an imaginary circle (*r* = 7.5 degrees of visual angle) around the fixation cross with six possible stimulus locations. After a variable stimulus duration the letter display was terminated by a 500-ms masking display containing six masks made from red and blue letter fragments. Then the screen turned black, and the participant could type in the letters that he or she had seen. In whole report trials either two or six red target letters were presented, while partial report trials featured two red target letters and four blue distractor letters. Displays with six target letters were shown for each of six stimulus durations (10, 20, 50, 80, 140, or 200 ms) while all other displays were shown for 80 ms. All trial types were intermixed and the stimuli in a given trial were chosen randomly without replacement from a set of 20 capital letters [ABDEFGHJKLMNOPRSTVXZ] written in the font Arial (broad) with a letter point size of 68 corresponding to 2.7 by 2.3 degrees of visual angle. The individual, multi-colored masks were 100 by 100 pixels to completely cover the letters. Participants were instructed to make an unspeeded report of all red letters they were “fairly certain” of having seen, that is to use all available information but refrain from pure guessing. Participants were informed of the accuracy of their reports (the probability that a reported letter was correct) after each block and they were encouraged to report as many red letters as possible but keep their reports within a specified accuracy range of 80–90% correct. The stimulus displays were presented on a 21″ EIZO CRT monitor at 100 Hz using the E-prime 1.1 software. All tests were run in a windowless room with standard lighting conditions, with participants seated in front of a monitor with a viewing distance of approximately 60 cm.

#### Estimation of TVA parameters

The number of correctly reported letters in each trial constituted the main dependent variable in the *CombiTVA* task. The parameter estimates can be extracted from the behavioral data by a maximum likelihood fitting procedure that is implemented in the publicly available Matlab toolbox *LIBTVA* (Kyllingsbæk, 2006; Dyrholm et al., 2011), and the mathematical interpretations of each of the TVA parameters are described in detail in the relevant publications. Briefly, assuming a particular set of parameters, one can calculate the probability for any possible outcome of a whole and partial report trial, and thereby, for a given individual, one can estimate the set of parameter values that maximize the probability of observing a given set of data. Storage capacity of visual short-term memory, *K*, was assumed to vary on a trial-by-trial basis and, thus, *K* for a particular trial was drawn from a probability distribution consisting of five free parameters (i.e., the probabilities that *K* = 1, 2,…, 5), where these probabilities summed to a number between 0 and 1, and the remaining probability up to a value of 1 was accounted for by the probability that *K* = 6. Thus, the *K* value reported here is the expected *K* given a particular probability distribution. The perceptual threshold, *t*_0_, was assumed to be drawn trial-by-trial from a normal probability distribution with two free parameters (mean and *SD*). The total visual processing speed, *C*, is characterized by only one free parameter (i.e., a constant) defined as the sum of all *v* values across all perceptual categorizations of all elements in the visual field:

$$C=\;\sum_{x\;\in \;S}\sum_{j\;\in \;R}v(x,i)$$

Although participants were instructed to distribute attention uniformly among the possible targets in the stimulus display, they might behave differently; so attentional weights (*w* values) were estimated individually for targets at each of the six stimulus locations (5 free parameters, as the sum of the 6 attentional weights was fixed at a value of 1). Furthermore, assuming that attentional weights of every target is equal to the weight of every other target for a given spatial location, and that the attentional weight of every distractor is similar to the weight of every other distractor on the same spatial location, the visual selectivity parameter, α (one free parameter), can be estimated by the ratio of the distractor and target attentional weights:

$$\alpha =w_{distractor}/w_{target}$$

In sum, the specific model used for the fitting procedure had a total of 14 free parameters.

#### TVA inclusion criteria

We set the following inclusion criteria: *t*_0_ estimation error < 30, *C* estimation error < 80, and relative attentional weight <0.8 on all six positions in the display. 325 of 347 data sets satisfied these criteria. The 22 excluded participants typically failed to follow instructions, for example by focusing primarily on only one of the target positions instead of the fixation cross, with the result that the attentional weight for that position would be >0.8 and the overall *K* estimate would be close to 1. These individuals covered most of the age range, but were predominantly in the 60s and 70s (Mean age = 63, Median age = 68).

### MR acquisition and analysis—diffusion tensor imaging

The data and processing scheme was performed as previously described (Westlye et al., 2012b). Imaging was performed on a 12-channel head coil on a 1.5-T Siemens Avanto scanner (Siemens Medical Solutions, Erlangen, Germany) at Oslo University Hospital, Rikshospitalet. For diffusion weighted imaging a single-shot twice-refocused spin-echo echo planar imaging sequence with the following parameters was used: repetition time (TR)/echo time (TE) = 8590/87 ms, *b*-value = 1000 s/mm^2^, voxel size = 2.0 × 2.0 × 2.0 mm, and 64 axial slices. The sequence was repeated twice with 10 *b* = 0 and 60 diffusion-weighted volumes per run.

DTI datasets were processed using the FMRIB Software Library (FSL) (Smith et al., 2004). Each volume was affine registered to the first *b* = 0 volume using FMRIB's linear image registration tool (FLIRT) (Jenkinson et al., 2002) to correct for motion and eddy currents. After removal of non-brain tissue, FA (Basser and Pierpaoli, 1996), eigenvector, and eigenvalue maps were computed by linearly fitting a diffusion tensor to the data. We defined RD as the mean of the second and third eigenvalue [(λ_2_ + λ_3_)/2] and MD as the mean of all three eigenvalues. FA volumes were skeletonized and transformed into a common space as employed in Tract Based Spatial Statistics (TBSS) (Smith et al., 2006). All volumes were non-linearly warped to the FMRIB58_FA template by use of local deformation procedures performed by FMRIB's non-linear image registration tool (FNIRT). Next, a mean FA volume of all subjects was generated and thinned to create a mean FA skeleton representing the centers of all common tracts. We thresholded and binarized the mean skeleton at FA > 0.2 to reduce the likelihood of partial voluming in the borders between tissue classes, yielding a mask of 1,20,770 voxels. Individual FA values were warped onto this mean skeleton mask by searching perpendicular from the skeleton for maximum FA values. Using maximum FA values from the centers of the tracts further minimizes confounding effects due to partial voluming (Smith et al., 2006). Similar warping and analyses were employed on MD, AD, and RD data, yielding skeletons sampled from voxels with FA > 0.20. DTI data were on average acquired 1.1 years (*SD* = 0.28) after TVA-based assessment was performed.

Mean DTI values were calculated within defined anatomical white matter regions and pathways by masking the TBSS skeletons with regions of interests (ROIs) based on digitalized probabilistic white matter and tractography atlases (Mori et al., 2005; Wakana et al., 2007; Hua et al., 2008). The ROIs included the body of corpus callosum (BCC), cingulum (CGC), corona radiata (CR), corticospinal tract (CST), external capsule (EC), fornix (FX), genu of corpus callosum (GCC), internal capsule (IC), inferior longitudinal fasciculus (ILF), posterior thalamic radiation (PTR), splenium of corpus callosum (SCC), superior fronto-occipital fasciculus (SFO), superior longitudinal fasciculus (SLF), sagittal stratum (SS), and the uncinate fasciculus (UNC).

### Statistical analysis

Relations between behavioral measures were analyzed with bivariate correlation analysis. To assess stability of the correlations across the adult life span (~20–80), the sample was split into two age groups with similar age spans (<50, ≥50). To describe age-related effects on means and standard deviations for each of the behavioral measures, the sample was split into six age groups of equal size (*n* = 54). For comparison across measures, all measures were transformed to standard scores and compared across the six age groups. Age effects on each of the TVA parameters were further analyzed with independent sequential linear regression models in which the TVA parameters were dependent variables, and age (first), and age^2^ (second) was entered as independent variables. The age-relation was considered to be quadratic if the *R*^2^ change was significant, and the age-relation was otherwise considered to be linear. The same set of analyses was repeated for the two age groups (<50, ≥50). We compared slopes across age groups by running linear regression analyses including the interaction between age and age group as independent variable. Associations between DTI indices (whole brain averaged values) and behavioral data was analyzed with partial correlation analyses, including first sex and time elapsed between DTI and TVA acquisition as covariates, and subsequently age and age^2^. The regional specificity was assessed in a similar way. Mediation analysis were done based on the criteria specified by Baron and Kenny (1986), as explained in the Results Section.

## Results

### Behavioral data and the effects of age

**Figure 2:**
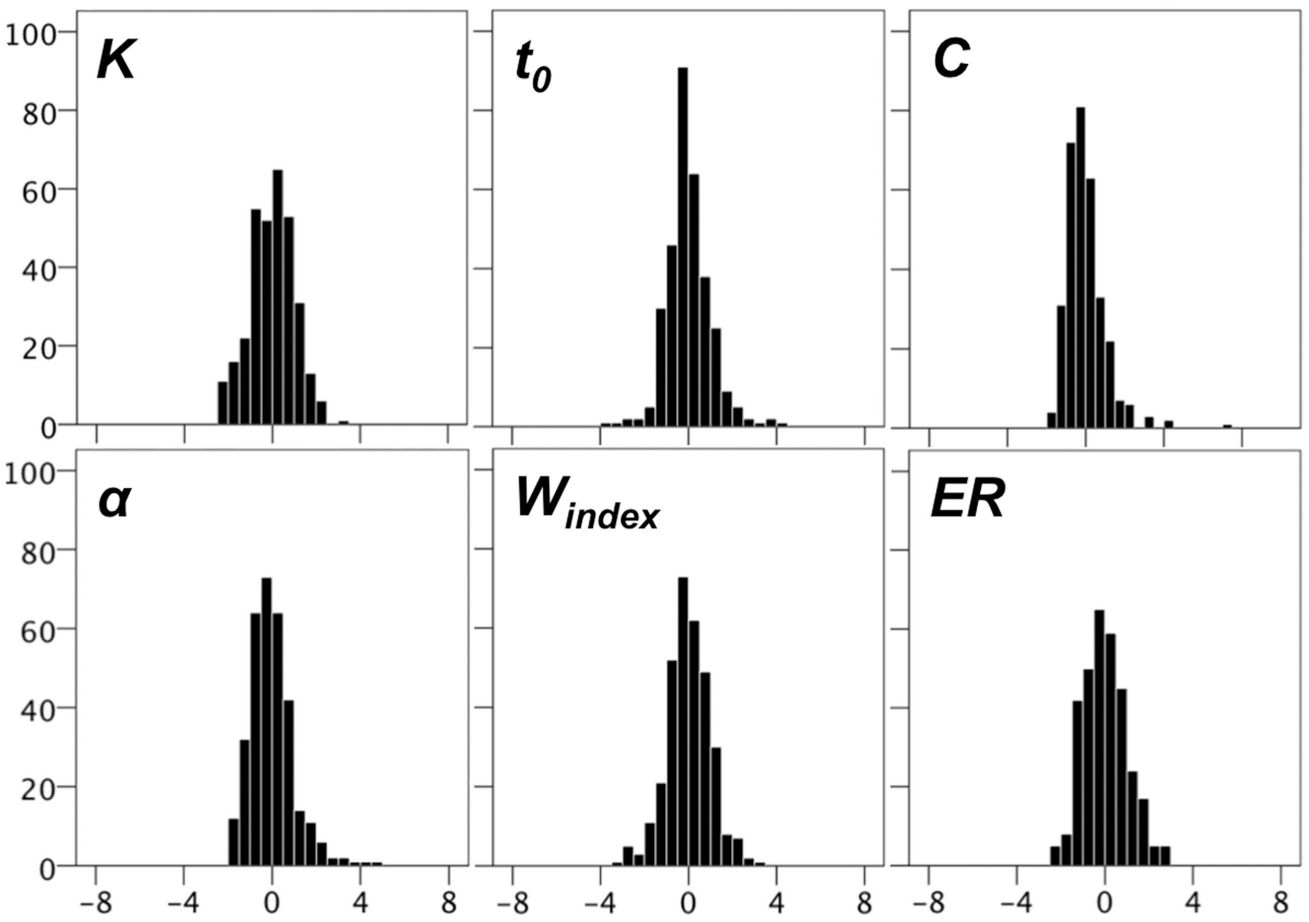
**Histograms for TVA parameters, and for error rates (ER) obtained from the *CombiTVA* paradigm**. The y-axes displays frequency and the x-axes displays z-values.

After applying inclusion criteria TVA parameters were close to normally distributed with almost all values within ±4 *SDs* when normalized across the whole sample. Figure 2 shows that there was a small number of remaining high *t*_0_, *C*, and α scores.

Results from a bivariate correlation analysis are shown in Table 1. *C* and *t*_0_ were uncorrelated whereas there was a strong correlation between *C* and *K*. The weight index and α were not strongly correlated with any of the other parameters. *DSS* and *g*F were strongly correlated with each other and had virtually identical associations with the TVA parameters. The pattern of correlation within TVA was generally stable across the age range in the present sample when compared between two age cohorts spanning approximately 30 years each. However, TVA parameter correlations with *DSS* and *g*F varied with age group. Notably, *K* and *C* correlations with *DSS* were substantially weaker in the old group, as was the α correlation with *g*F. In contrast, the *t*_0_-*g*F correlation was clearly stronger in the old group than in the young group.

**Table 1:** Bivariate correlations between TVA parameters, *DSS*, and *g*F, for the full sample, and split into age groups (<50, ≥50). ^*^p < 0.05, ^**^p < 0.01.

	***K***	***t*_0_**	***C***	***α***	***W*_index_**	***DSS***
**FULL SAMPLE (*n* = 325)**						
*K*						
*t_0_*	−0.21^**^					
*C*	0.53^**^	0.03				
α	−0.10	0.14^*^	−0.08			
*w*_index_	−0.14^*^	0.02	−0.11^*^	−0.03		
*DSS*	0.38^**^	−0.28^**^	0.41^**^	−0.17^**^	−0.06	
*g*F	0.38^**^	−0.28^**^	0.42^**^	−0.22^**^	−0.01	0.65^**^
**AGE < 50 (*n* = 147)**						
*K*						
*t*_0_	−0.31^**^					
*C*	0.49^**^	0.04				
α	−0.11	0.17^*^	−0.14			
*w*_index_	−0.22^**^	0.05	−0.15	−0.08		
*DSS*	0.44^**^	−0.25^**^	0.45^**^	−0.19	−0.08	
*g*F	0.33^**^	−0.15	0.30^**^	−0.26^**^	−0.05	0.57^**^
**AGE ≥ 50 (*n* = 178)**						
*K*						
*t*_0_	−0.10					
*C*	0.51^**^	0.11				
α	−0.03	0.09	0.07			
*w*_index_	−0.08	0.00	−0.08	−0.01		
*DSS*	0.24^**^	−0.24^**^	0.20^**^	−0.12	−0.06	
*g*F	0.27^**^	−0.30^**^	0.37^**^	−0.13	0.04	0.57^**^

The total sample spans about six decades of adult life and Table 2 shows descriptive statistics for the behavioral data across the entire age range split up in six equally sized age groups covering about one decade each.

**Table 2:** **Descriptive statistics for complete sample, split into six equally sized age groups**.

	**Age group 1**	**Age group 2**	**Age group 3**	**Age group 4**	**Age group 5**	**Age group 6**
Cohort size	54	54	55	54	54	54
Age range (M)	20–27 (23.6)	27–42 (36.3)	43–52 (47.8)	52–60 (55.8)	60–68 (64.3)	68–81 (73.4)
Sex (F/M)	41/13	39/15	35/20	32/22	36/18	36/18
EIQ	121.7 (11.2)	118.6 (9.6)	117.4 (10.9)	121.1 (7.9)	121.9 (8.3)	124.0 (11.6)
DSS	69.3 (9.9)	63.8 (9.7)	59.8 (12.3)	55.5 (9.2)	52.7 (10.3)	49.4 (10.4)
*K*	3.47 (0.7)	3.30 (0.8)	3.11 (0.8)	3.01 (0.9)	2.78 (0.8)	2.60 (0.8)
*t*_0_	16.07 (10.2)	17.07 (10.0)	17.94 (12.5)	17.05 (14.2)	22.14 (14.9)	27.38 (17.3)
*C*	60.1 (24.9)	58.05 (29.8)	52.27 (19.2)	46.51 (17.3)	42.07 (21.9)	39.50 (19.2)
*α*	0.70 (0.3)	0.90 (0.4)	0.91 (0.4)	0.92 (0.4)	1.00 (0.4)	0.88 (0.4)
*W*_index_	0.51 (0.1)	0.53 (0.1)	0.53 (0.1)	0.53 (0.1)	0.53 (0.1)	0.52 (0.1)
Error rate	0.26 (0.1)	0.27 (0.1)	0.32 (0.1)	0.32 (0.1)	0.34 (0.1)	0.31 (0.1)

**Figure 3:**
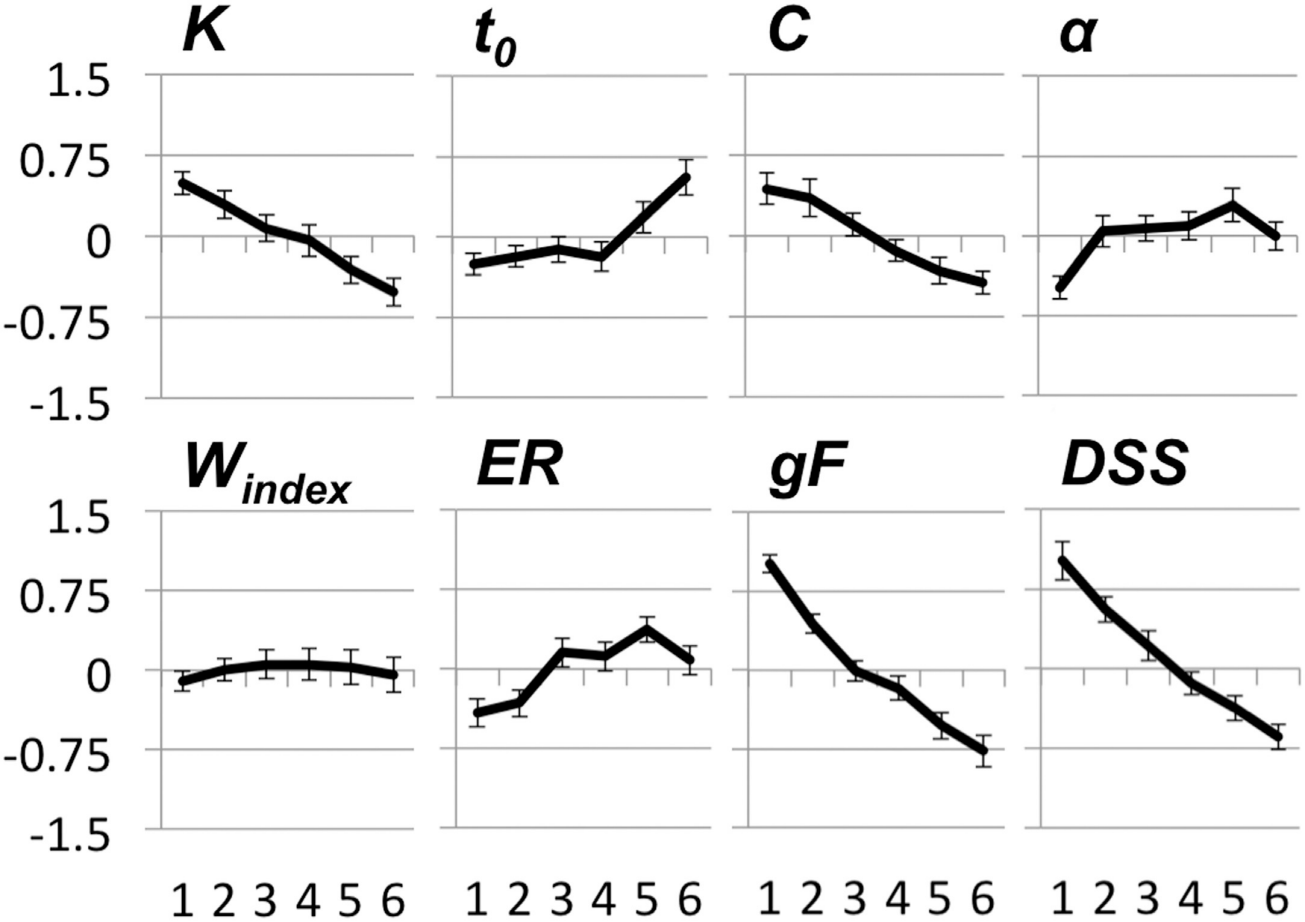
**Displays effects of age on TVA parameters, error rate (ER) from the *CombiTVA* paradigm, fluid intelligence (*g*F) and digit-symbol substitution (*DSS*)**. The y-axes displays z-values and the x-axes refers to age groups. Age groups are the same as in Table 2. Error bars are standard error of the mean. See text for details.

Of note, estimated IQ was more than one standard deviation above the age-adjusted population mean for all age groups. The highest IQs were estimated for the two oldest groups. To compare age effects across measures we transformed all TVA parameters and the *DSS* score to standardized scores across all subjects and plotted these for the six age groups (see Figure 3).

As evident from Figure 3, *K*, the capacity of visual short-term memory, and *C*, the speed of visual processing, seem to decline linearly across the age range, although not as steeply as *DSS* and *g*F. *t*_0_, the perceptual threshold, and α, the parameter for attentional selectivity, have more complex age trajectories; the α parameter increases in the early part of the age range and is relatively stable thereafter, whereas *t*_0_ displays an opposite pattern of age effects with early stability and late increase. The weight index seems to be unrelated to age.

**Figure 4:**
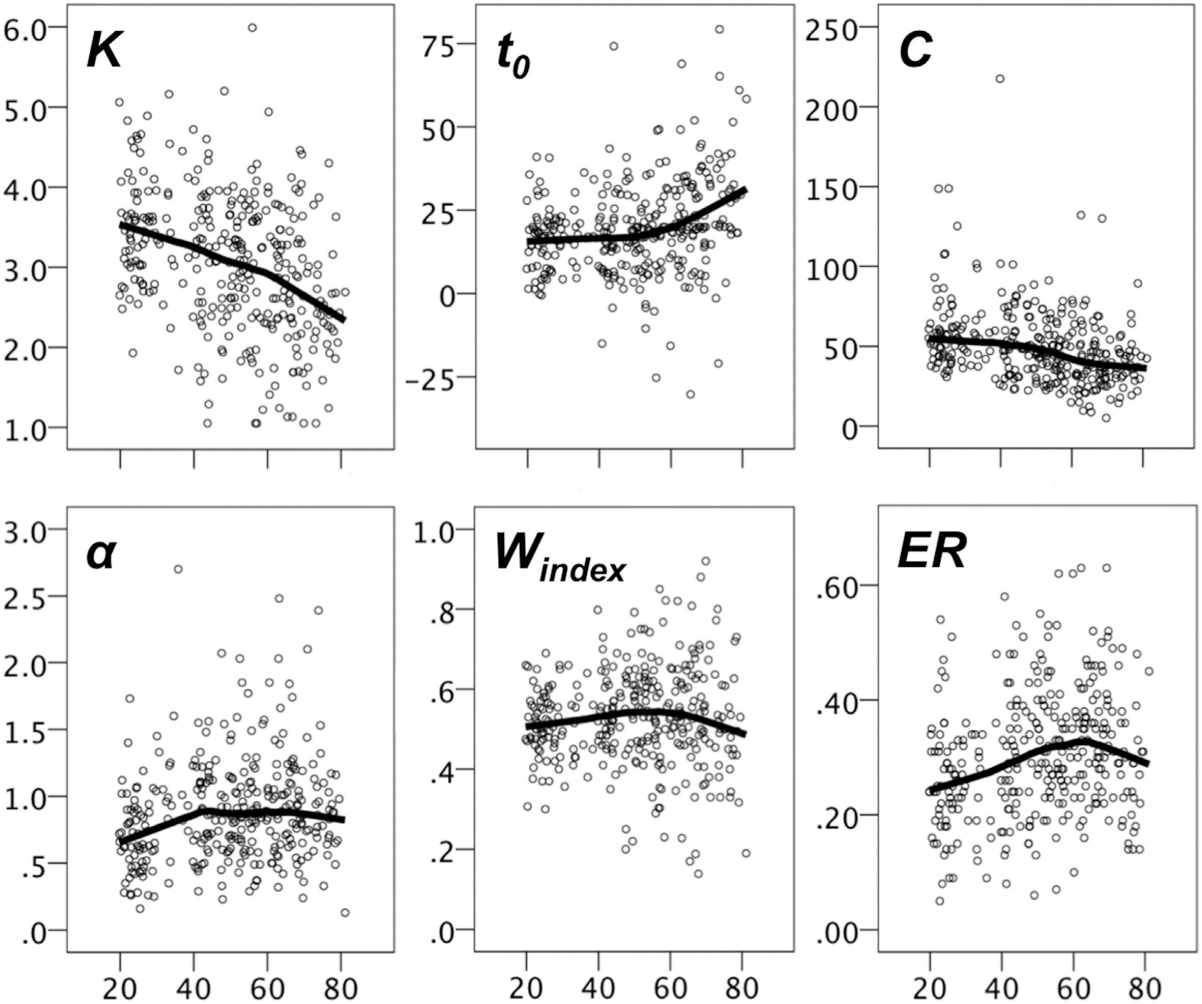
**Scatter plots with LOESS fits for TVA parameters**. The y-axes displays the parameter values and the x-axes displays age.

Figure 4 shows scatter plots of the TVA parameter data with LOESS fits. The magnitude of the age effect for each measure, and whether the age trends were best characterized by linear or quadratic fits were assessed with sequential linear regression tests. As evident from Table 3A, all parameters were significantly associated with age, but only *t*_0_ and α had any increase in explained variance for the quadratic model as evidenced by the *R*^2^ change in a sequential regression model (*p* = 0.001 and *p* = 0.008, respectively). When the same set of analyses were repeated for the two age groups (<50, ≥50), all age trends were linear (i.e., non-significant *R*^2^ change, see Table 3B). For the young group, the parameters most strongly associated with age were *K* and α. For the old group, *K*, and particularly *t*_0_, were most strongly related to age, the latter with a *R*^2^ value that went beyond the *R*^2^ of *DSS* and *g*F. To test whether age slopes were different across age groups, we ran independent linear regression analyses for each of the TVA parameters, including age group, age, and their interaction (i.e., age group × age) as independent variables, and these analyses revealed that the interaction term was significant for *t*_0_, *t* = 3.15, *p* = 0.002, and for α, *t* = −2.23, *p* = 0.027, but not for *K* (*p* = 0.56), and *C* (*p* = 0.86).

**Table 3A:** **Based on sequential linear regression model tests**.

**Y**	**Model**	**Equation of Y**	***F* value**	***R***	***R*^2^ (%)**	***R*^2^ change**
*K*	Linear	−0.018x + 3.95	*F*_(1, 23)_ = 46.8^***^	0.36	12.7	0.1
*t*_0_	Quadratic	−0.64x + 0.009x^2^ + 27.3	*F*_(2, 322)_ = 16.0^***^	0.25	6.1	2.9
*C*	Linear	−0.446x + 72.13	*F*_(1, 323)_ = 37.4^***^	0.32	10.4	0.0
α	Quadratic	0.024x + (−0.0002x^2^) + 0.28	*F*_(2, 322)_ = 7.9^**^	0.16	2.6	2.1
*DSS*	Linear	−0.398x + 78.4	*F*_(1, 275)_ = 94.9^***^	0.51	25.7	0.0
*g*F	Linear	0.001x + 0.23	*F*_(1, 318)_ = 173.0^***^	0.59	35.2	0.1

R^2^ change refers to increase in R^2^ when a quadratic term is added to the model. F- and p-values presented for the linear model except in case of t_0_ and α, for which the R^2^ change was significantly improved after adding the quadratic age term. ^*^p ≤ 0.01,

** *p ≤ 0.001*,

*** *p ≤ 0.0001*.

**Table 3B:** **Based on sequential linear regression model tests, split on ages <50 and ≥50**.

**Y**	**Equation of Y**	***F* value**	***R***	***R*^2^ (%)**	***R*^2^ change**
**AGE < 50**					
*K*	−0.020x + 3.99	*F*_(1, 146)_ = 9.25^*^	0.25	6.0	0.5
*t*_0_	0.106x + 13.65	*F*_(1, 146)_ = 1.26	0.09	0.1	0.0
*C*	−0.276x + 67.21	*F*_(1, 146)_ = 1.62	0.11	1.1	0.4
*α*	0.009x + 0.53	*F*_(1, 146)_ = 7.83^*^	0.23	5.1	1.7
*DSS*	−0.354x + 77.2	*F*_(1, 105)_ = 8.77^*^	0.28	7.7	0.1
*g*F	−0.037x + 1.84	*F*_(1, 146)_ = 47.9^***^	0.50	25.0	0.0
**AGE ≥ 50**					
*K*	−0.026x + 4.48	*F*_(1, 176)_ = 12.2^**^	0.26	6.5	0.0
*t*_0_	0.621x + (−17.76)	*F*_(1, 176)_ = 21.67^***^	0.33	11.0	0.0
*C*	−0.326x + 63.78	*F*_(1, 176)_ = 3.56	0.14	2.0	0.2
*α*	0.002x + 1.05	*F*_(1, 176)_ = 0.28	0.04	0.2	0.9
*DSS*	−0.325x + 73.5	*F*_(1, 168)_ = 12.0^**^	0.26	6.7	0.0
*g*F	−0.031x + 1.49	*F*_(1, 173)_ = 13.0^***^	0.27	7.0	0.0

*R^2^ change refers to increase in R^2^ when a quadratic term is added to the model. F- and p-values presented for the linear model. Age ranges = 19–49, n = 147, and 50–81, n = 178*.

* *p ≤ 0.01*,

** *p ≤ 0.001*,

*** *p ≤ 0.0001*.

### DTI indices and the effects of age

**Figure 5:**
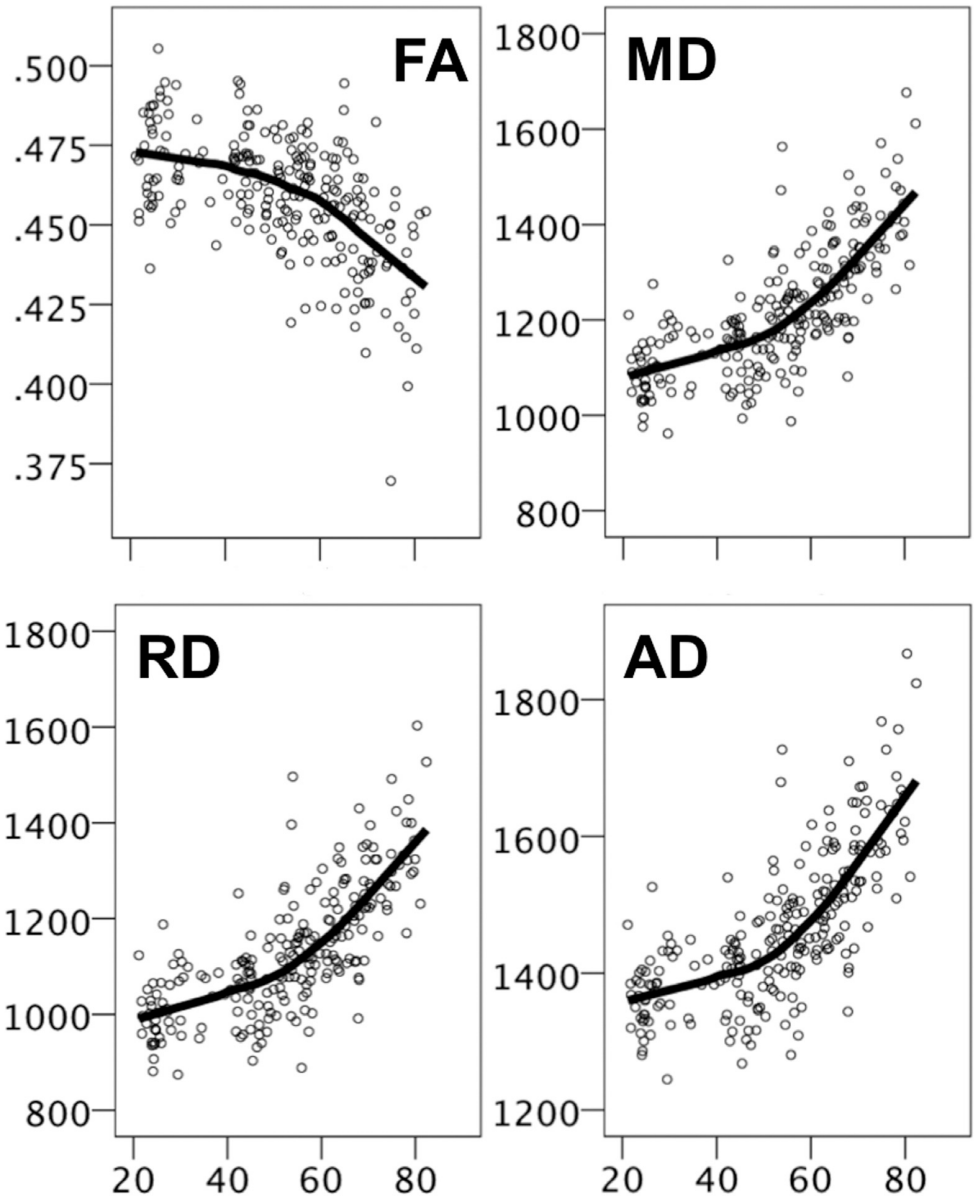
**Scatter plots with LOESS fits for DTI indices**. The y-axes displays FA values and diffusivity values (mm^2^/second) for MD, RD, and AD, and the x-axes displays age.

For the DTI indices, there were strong age effects on all parameters, relatively weaker for FA than for the diffusivity measures, which were almost perfectly correlated with each other (all *r*'s ≥ 0.99), even after controlling for age. Figure 5 displays scatter plots with LOESS fits for each of the DTI parameters. Age effects were non-linear, with steeper age-related effects from around mid-life. As for the behavioral data, age trends were approximately linear when the sample was split at age 50 (data not shown), all significant with the exception of FA in the young group (*p* = 0.08).

Results from linear regressions assessing age effects on DTI values averaged across the WM skeleton and 15 separate ROIs are displayed in Table 4.

**Table 4:** **Based on linear regression model tests with DTI measures as dependent variables, and sex, age, and age^2^ as independent variables**.

	**Avg**.	**BCC**	**CGC**	**CR**	**CST**	**EC**	**FX**	**GCC**	**IC**	**IFO**	**PTR**	**SCC**	**SFO**	**SLF**	**SS**	**UNC**
**FA**																
*R*^2^	0.38	0.30	0.18	0.32	0.08	0.19	0.44	0.59	0.12	0.05	0.27	0.15	0.23	0.18	0.18	0.17
*F*	53.6	37.5	19.4	41.2	7.0	20.6	68.0	45.9	12.1	4.5	31.1	15.1	25.7	18.3	18.4	17.1
*p*	^***^	^***^	^***^	^***^	^**^	^***^	^***^	^***^	^***^	^*^	^***^	^***^	^***^	^***^	^***^	^***^
**MD**																
*R*^2^	0.62	0.35	0.46	0.43	0.23	0.26	0.38	0.46	0.37	0.39	0.19	0.23	0.34	0.49	0.52	0.09
*F*	142.0	46.9	72.4	64.1	25.3	30.1	52.7	74.3	50.7	54.8	20.1	25.7	43.7	82.0	94.9	8.1
*p*	^***^	^***^	^***^	^***^	^***^	^***^	^***^	^***^	^***^	^***^	^***^	^***^	^***^	^***^	^***^	^***^
**AD**																
*R*^2^	0.61	0.35	0.45	0.34	0.10	0.24	0.35	0.40	0.40	0.23	0.12	0.17	0.35	0.45	0.45	0.06
*F*	136.4	45.4	70.9	44.8	9.7	26.7	46.4	58.6	57.6	25.3	12.6	17.6	46.1	69.6	71.1	5.1
*p*	^***^	^***^	^***^	^***^	^***^	^***^	^***^	^***^	^***^	^***^	^***^	^***^	^***^	^***^	^***^	^*^
**RD**																
*R*^2^	0.62	0.35	0.35	0.43	0.24	0.23	0.39	0.47	0.38	0.35	0.21	0.25	0.34	0.48	0.54	0.10
*F*	139.8	47.2	46.6	66.0	27.4	25.9	55.3	77.3	52.0	46.8	23.0	28.0	45.0	80.6	99.5	9.9
*p*	^***^	^***^	^***^	^***^	^***^	^***^	^***^	^***^	^***^	^***^	^***^	^***^	^***^	^***^	^***^	^***^

*Degrees of freedom for F = 3, 259 for all analyses. Avg, average parameter value across TBSS skeleton; BCC, body of corpus callosum; CGC, cingulum; CR, corona radiate; CST, corticospinal tract; EC, external capsule; FX, fornix; GCC, genu of corpus callosum; IC, internal capsule; IFO, inferior longitudinal fasciculus; PTR, posterior thalamic radiation; SCC, splenium of corpus callosum; SFO, superior fronto-occipital fasciculus; SLF, superior longitudinal fasciculus; SS, sagittal stratum; UNC, uncinate fasciculus*.

* *p < 0.01*,

** *p < 0.001*,

*** *p < 0.0001*.

For FA, age effects were most pronounced in the corpus callosum, the corona radiata, and the fornix. For the diffusivity measures the effects of age were on average larger, and the ROIs with the most pronounced effects were the corpus callosum, the cingulum, the corona radiata, the superior longitudinal fasciculus, and the sagittal stratum.

### Associations between behavioral data and DTI indices

#### Associations between behavioral data and DTI indices, and effects of controlling for age

The association between behavioral measures (TVA parameters, *DSS, g*F) and DTI parameters (FA and MD averaged across the skeleton) were done with partial correlation analyses with effects of sex and difference in time elapsed between TVA and DTI acquisition partialled out. RD and AD was not included since these correlated almost perfectly with MD. For the complete sample of 229 participants who had concurrent TVA and DTI data, it was revealed that all behavioral measures, except α, correlated significantly with both average FA and average MD, with *r*'s ranging from 0.17 (*K*-FA) to −0.53 (*g*F-MD). After controlling for age, no correlation with FA remained significant. However, the correlations between *t*_0_ and MD, and between *g*F and MD, were still significant (*r* = 0.18, *p* < 0.01, *df* = 226 for *t*_0_, *r* = −0.18, *p* < 0.01, *df* = 222 for *g*F). Including the quadratic age term as an additional confounder further reduced the *t*_0_-MD correlation (*r* = 0.13, *p* = 0.05, *df* = 225), but somewhat strengthened the *g*F-MD correlation (*r* = −0.20, *p* < 0.01, *df* = 221), and also made the *DSS*-MD correlation nominally significant (*r* = −0.15, *p* < 0.05, *df* = 196).

Given the finding of a mixture of linear and non-linear age effects, and that most effects were linear within the age groups defined by split at age 50, we reran the partial correlation analyses for each group separately. The results are displayed in Table 5.

**Table 5:** **Based on partial correlations controlling for sex and time elapsed between behavioral testing and DTI scanning**.

	***K***	***t*_0_**	***C***	***α***	***DSS***	***g*F**
**AGE < 50**						
FA	0.15	−0.11	0.31^**^	−0.09	0.35^**^	0.23^*^^#^
MD	0.0	0.03	−0.05	0.09	−0.25^*^^#^	−0.21^*^^#^
**AGE ≥ 50**						
FA	0.02	−0.15	−0.04	0.14	0.15	0.14
MD	−0.20^*^^#^	0.35^***^	−0.11	−0.07	−0.34^***^	−0.34^***^

* *p ≤ 0.05*,

** *p ≤ 0.01*,

*** *p ≤ 0.001*,

# *non-significant after controlling for age and age^2^*.

As can be seen in the table, *C, DSS*, and *g*F were correlated with FA, and *DSS* and *g*F with MD for the young participants, but only the *C*-FA and *DSS*-FA correlations remained when controlling for age and age^2^ (*r* = 0.29, *p* = 0.005 for *C*-FA, *r* = 0.32, *p* = 0.008 for *DSS*-FA). For the old group *K, t*_0_, *DSS*, and *g*F correlated significantly with MD, but the *K*-MD correlation did not survive controlling for age and age^2^. The remaining correlations were reduced but still significant (*r* = 0.20, *p* = 0.019 for *t*_0_-MD, *r* = −0.20, *p* = 0.022 for *DSS*-MD, and *r* = −0.27, *p* = 0.002 for *g*F-MD). There were no significant correlations with FA in the old group.

#### Are effects of age on t_0_ mediated by differences in WM diffusivity?

In order to test whether WM diffusivity mediates the correlation between *t*_0_ and age in the old group, we assessed the patterns of correlations according to Baron and Kenny's (1986) four criteria for mediation. These state that significant associations between (1) the independent variable (i.e., age) and the dependent variable (i.e., *t*_0_), and (2) between the independent variable and the mediator (i.e., average MD) must be established. In the old group, these criteria were satisfied since age was significantly correlated with both *t*_0_ and MD. Furthermore, it needs to be established that (3) the mediator is associated with the dependent variable after controlling for the independent variable. This criterion was also satisfied since the *t*_0_-MD correlation was significant even after controlling for age and age^2^. Finally, it needs to be shown that (4) the association between the independent variable and the dependent variable is weakened when controlling for the potential mediator. To test this, we performed partial correlation analyses with age and *t*_0_, controlling first for sex and differences in elapsed time between TVA and DTI acquisition. Zero-order *r* = 0.33 (*r*^2^ = 11.0%), whereas the partial correlation was *r* = 0.30 (*r*^2^ = 9.0%). Controlling also for average MD further reduced the partial correlation to *r* = 0.12 (*r*^2^ = 1.4%). Thus, for participants aged 50 years and above controlling for average MD reduced the variance in *t*_0_ explained by age with 84.4%.

#### Are associations of DTI indices on multicomponent measures mediated by TVA parameters?

To test whether the relation between DTI parameters, processing speed, and fluid intelligence were mediated by TVA parameters, we assessed the correlations between them according to Baron and Kenny's (1986) four criteria for mediation.

In the young group, we examined whether the correlation between FA and *DSS* was mediated by *C*. FA was correlated with *DSS* and *C*, also after controlling for age and age^2^ (criteria 1 and 2). The correlation between *C* and *DSS* was significant after controlling for age and age^2^, and FA (*r* = 0.44, *p* < 0.0005) (criterion 3). To test whether the association between the independent variable (i.e., FA) and the dependent variable (i.e., *DSS*) was weakened when controlling for the potential mediator (i.e., *C*) (criterion 4), we performed partial correlation analyses with FA, *DSS*, controlling for sex and difference in elapsed time between TVA and DTI acquisition, and *C*. FA correlation with *DSS* was reduced from *r* = 0.35 (*r*^2^ = 12%) to *r* = 0.24 (*r*^2^ = 6%), and was no longer significant. Thus, controlling for *C* reduced the variance in *DSS* explained by FA with about 50%.

In the old group, we examined whether the correlations between MD and *DSS*, and between MD and *g*F were mediated by *t*_0_. For the MD-*DSS* correlation, criteria 1 and 2 were satisfied because MD was significantly correlated with both *DSS* and *t*_0_, also after controlling for age and age^2^. However, the criterion 3 was not satisfied since *t*_0_was not significantly correlated with *DSS* after controlling for MD (*r* = −0.16, *p* = 0.073). The correlation between MD and *DSS* was reduced from *r* = −0.34 (*r*^2^ = 12%) to *r* = −0.27 (*r*^2^ = 7%), when *t*_0_ was partialled out (criterion 4). This corresponds to a reduction in explained variance of about 42%. Thus, only three of the four criteria were satisfied.

For the MD-*g*F correlation, criteria 1 and 2 were satisfied since MD was significantly correlated with both *g*F and *t*_0_. The *t*_0-*g*_*F* correlation was significant after controlling for age, age^2^, and average MD (criterion 3), and the correlation between MD and *g*F was reduced from *r* = −0.34 (*r*^2^ = 12%) to *r* = −0.28 (*r*^2^ = 8%) when *t*_0_ was partialled out (criterion 4). This corresponds to a reduction of variance in *g*F explained by MD with about 33%.

#### Analysis of regional specificity of behavior-DTI correlations

To identify potential ROI-specific associations between behavioral measures and WM tracts that were significant at the whole-skeleton level, we performed partial correlation analyses with FA, *C, DSS*, and *g*F in the data from young participants, and with MD, *t*_0_, *DSS*, and *g*F in the data from the old participants with all ROIs, including also ROI subsections (i.e., anterior, posterior, etc.) when available. The results are displayed in Tables 6, 7.

**Table 6:**
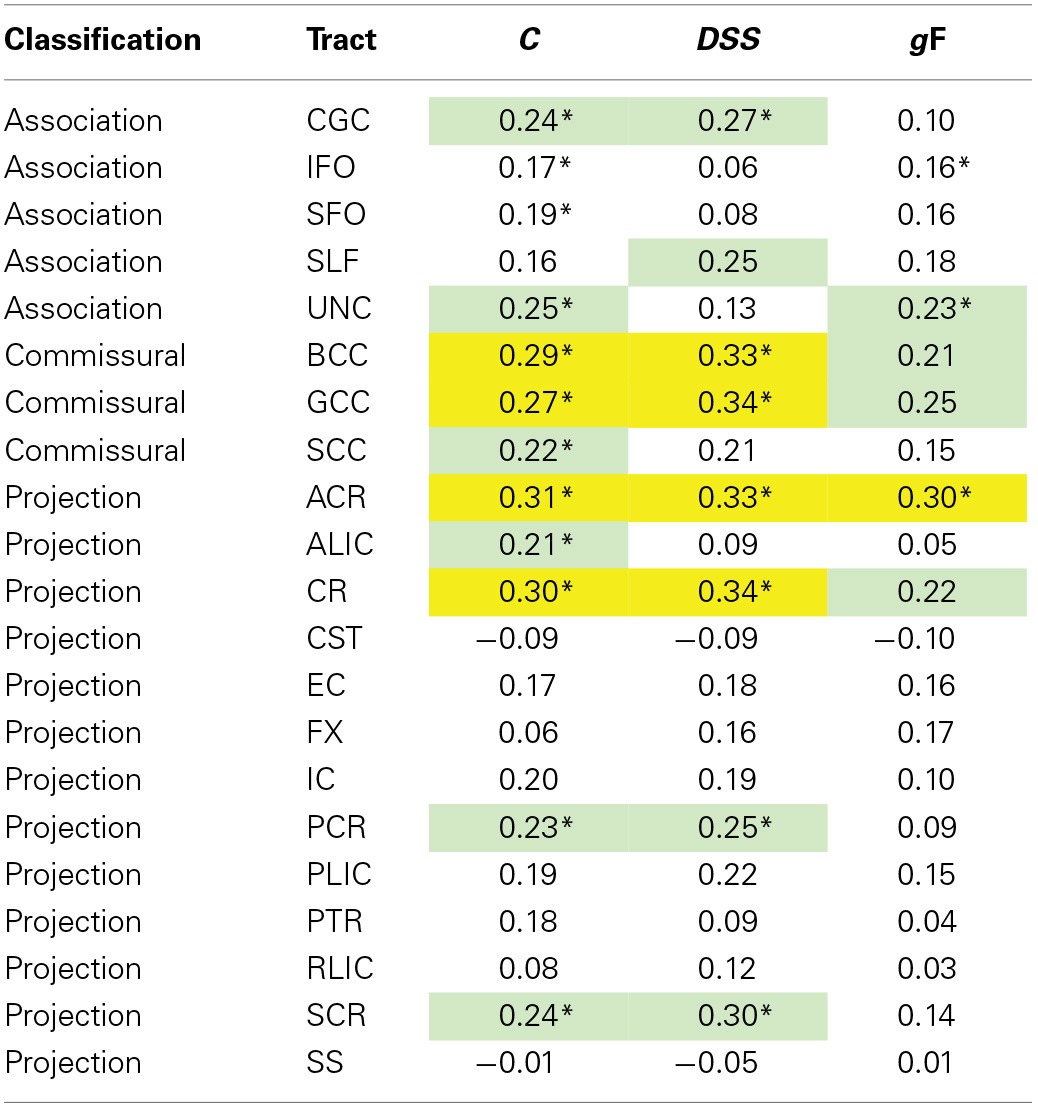
**Partial correlations between ROI FA values and behavioral measures, controlling for sex, and time elapsed between TVA and DTI acquisition**.

Age < 50. Green, p ≤ 0.05; yellow, p ≤ 0.01. ^*^Significant after adding age and age^2^ as control variables.

**Table 7:**
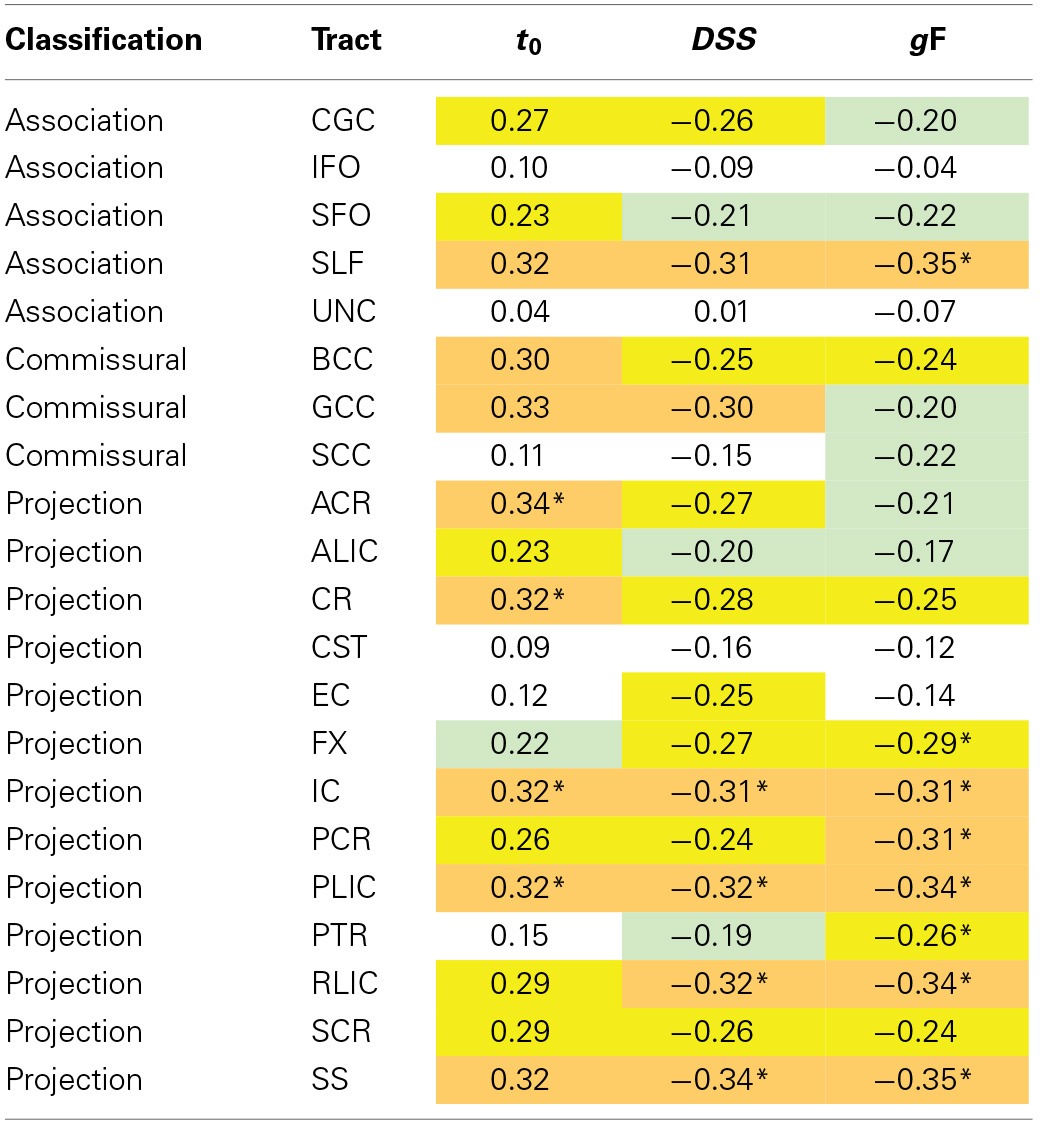
**Partial correlations between ROI MD values and behavioral measures, controlling for sex, and time elapsed between TVA and DTI acquisition**.

Age ≥ 50. Green, p ≤ 0.05; yellow, p ≤ 0.01; orange, p ≤ 0.001. ^*^Significant after adding age and age^2^ as control variables.

In FA-related correlations the most prominent ROIs were the corpus callosum and the corona radiata. The power to reveal significant differences between correlations was limited. However, Steiger's *Z* test analyses (Steiger, 1980) revealed that the correlation between *C* and the anterior corona radiata, was larger than the correlation between *C* and the CST (*p* < 0.01, one-tailed), and *C* and the SLF, *C* and the FX, and *C* and the RLIC (*p* < 0.05, one-tailed), but was not significantly different from the other correlations involving *C*. Results were similar after adding age and age^2^ as control variables, and similar across behavioral traits. The MD-related correlations tended to be stronger and the sample size was also larger. The most prominent ROIs were the internal capsule, the sagittal stratum, the corona radiata, the superior longitudinal fasciculus, and the corpus callosum. Within the internal capsule, the posterior limb and the retrolenticular part were most strongly associated with behavior. Within the corona radiata, the anterior part had the largest correlations. Steiger's *Z* test analyses (Steiger, 1980) revealed that significant *t*_0_-ROI correlations were not significantly different from each other (*p* > 0.05, one-tailed), but was significantly different from *t*_0_-ROI correlations that were non-significant in the primary analysis (*p* < 0.01, one-tailed). The pattern of correlation appeared quite similar for the three behavioral measures included. After adding age and age^2^ as control variables, only partial correlations involving CR and IC remained significant for *t*_0_. The *DSS* and *g*F correlations were more resistant to controlling for age, particularly for projection fibers.

## Discussion

The main purpose of the present study was to characterize effects of age on parameters derived from TVA-based assessment, and on brain structural connectivity as defined by DTI-based indices of WM microstructure. Furthermore, we aimed to investigate the extent to which the age-related effects on TVA parameters were associated with age-related differences in DTI indices, and specifically whether it can be shown that DTI indices mediate effects of age on TVA parameters, and whether TVA parameters can be shown to mediate the relationship between DTI indices and age-related decline in *DSS* and *g*F.

### TVA parameters and age

The four TVA parameters, *K*, *t*_0_, *C*, α, were all significantly associated with participant age. *K* was characterized by a linear decline from an average at about 3.7 objects at age 20 to about 2.5 objects in the 80s (~32%). *C*, the speed of processing, revealed a similar linear age-related trend with a decline from about 65 items/second at the age of 20 to about 40 items/second by the age of 80 (~38%). The age-effect on *t*_0_ was different: There was a modest increase with age until the 50s (about 1 ms, or ~6%), but from here to the age of 80, *t*_0_ increased with another 10 ms (~59%). In line with this observation, *R*^2^ was significantly improved when the quadratic age term was included in the model. The age effect on the *α* parameter was more complex. This parameter started to increase already in the 20s, continued to increase until the early 40s and was relatively stable thereafter, but with a trend toward an improvement for the oldest participants. As for *t*_0_, *R*^2^ for the *α* parameter was significantly improved when the quadratic age term was included in the model. The age trends for *DSS* and *g*F were linear and relatively strong compared to those of the TVA parameters.

The descriptive properties of the TVA parameters as measured in the present study correspond well to those reported from other samples (Vangkilde et al., 2011; McAvinue et al., 2012; Habekost et al., 2014). The effects of participant age were also broadly comparable to those reported in prior studies (McAvinue et al., 2012; Habekost et al., 2013). Comparison with the results of McAvinue et al. (2012) is of particular interest since the age ranges overlap substantially between studies and since the paradigms used were identical. The age effects on *K*, and especially on *C* were weaker in the present study than those reported by McAvinue and colleagues. For *K* this seems to be due to the combination of somewhat lower estimates for the youngest participants (i.e., 20s and 30s) in the present study as compared to the Irish sample (~3.4 vs. ~3.5, respectively), and higher scores in the older part (i.e., 60s and 70s) of the sample (~2.7 vs. ~2.5, respectively). For *C* it seems that the relatively small age effect is due to high scores for the old participants (~41 vs. ~34, respectively). Age effects on *t*_0_ and *α* were of similar magnitude across studies, and interestingly, both studies revealed similar age trajectories also for these parameters: Early increase followed by later stability for *α*, early stability and later increase for *t*_0_. This led us to rerun the linear regressions with two age groups split at 50 years of age. This analysis revealed that the age effect on *α* was relatively larger for participants under the age of 50, whereas *R*^2^ was relatively larger for *t*_0_ for participants above the age of 50. In fact, for this age range, *t*_0_ changes were the dominant pattern, with an effect beyond what was observed for *DSS* and even *g*F. Age effects (i.e., slopes) on the *t*_0_ and *α* parameters were significantly different across age groups, as confirmed by linear regression analysis where the age group × age interaction term was included. For *K, C*, and *DSS* the effect size was similar for the two age groups. The pattern of bivariate correlations indicates the relative independence of the TVA parameters with the exception of the *K-C* association replicated in most TVA-based studies (Bundesen and Habekost, 2008; Habekost et al., 2014) and the differential age trajectories provide further support for the notion of parameter independence.

The parameter for attentional selectivity, *α*, was best described by a quadratic term: the age-related effect was mainly seen early in adult life. This finding may suggest only a partial fit with the frontal aging hypothesis, which posits that cognitive processes that are supported by frontal lobe functions, such as executive attention, should show decline at an earlier age, and that this decline should also be of greater magnitude than for cognitive processes supported by other brain regions (West, 1996; Verhaeghen and Cerella, 2002). Our results support the former prediction, but not the latter. *K* and *t*_0_, which arguably represent lower level, or more basic visual attention capacity functions, showed age-related effects of greater magnitude. This pattern of results may indicate that more peripheral functional systems are becoming less efficient in the aging brain, while central functions, that may be involved in compensation or scaffolding processes are relatively more preserved (Madden, 2007; Park and Reuter-Lorenz, 2009).

### DTI parameters and age

White matter integrity, as measured by DTI indices, declines in normal aging (Westlye et al., 2010; Madden et al., 2012; Salami et al., 2012), and the present results fit well with prior research in this field by revealing decreasing FA and increasing MD with increasing age. In some studies, it has also been shown that RD increases relatively more with age than AD, suggesting that WM tract disconnection is driven preferentially by myelin changes. The present results did not support this since AD and RD age trajectories were similar and the measures almost perfectly correlated. The high correlation between diffusivity indices does not seem to merely reflect the large age range used in the present study as correlations were essentially unaffected by partialling out age, and was similar for the age groups employed. Thus, the effects may be equally driven by differences in myelin and in axonal integrity. The literature is less clear with regards to the regional pattern of age-related effects. Several organizational schemes have been presented, including the idea that anterior regions deteriorate earlier than posterior regions (Pfefferbaum et al., 2000), and the idea that the regions last to be myelinated are the first to be demyelinated (also known as the retrogenesis hypothesis) (Bartzokis, 2004). In the present results, there were strong effects for some of the anterior tracts (e.g., the GCC) and for tracts that project to and from anterior regions (e.g., the SLF and CGC), but also significant effects in posterior tracts (e.g., SS and IC). The present results are consistent with a relatively widespread WM-based disconnection of brain networks in healthy aging.

### Mediation of age effects on TVA parameters

In the present study we aimed to assess the extent to which DTI measures mediated age effects on TVA parameters. According to Baron and Kenny's (1986) four criteria for mediation, significant associations between (1) the independent variable (i.e., age) and the dependent variable (i.e., a TVA parameter), and (2) between the independent variable and the mediator (i.e., a DTI index) must be established. For the complete sample these two criteria were satisfied since age was associated with differences in *K*, *t*_0_, *C*, and *α*, and also with averaged FA and MD. Following Baron and Kenny, it needs to be established that (3) the mediator is associated with the dependent variable after controlling for the independent variable, and (4) that the association between the independent variable and the dependent variable is weakened when controlling for the potential mediator. In the full sample, the only significant TVA-DTI association that resisted statistically controlling for age and age^2^ was the MD-*t*_0_ correlation. However, this correlation was almost entirely due to effects in the old group, and we therefore assessed mediation effects in this group alone. In the old subsample, all four criteria were satisfied for the age-*t*_0_ association, since MD was significantly correlated with *t*_0_ even after controlling for age, and since controlling for MD clearly reduced the correlation between age and *t*_0_. It could also be the case that FA mediated the association between age and *C* since the FA-*C* correlation was significant after controlling for age, but the age-*C* correlation was probably too weak to establish such an effect.

### The neuroanatomy of TVA

NTVA is a general neurophysiological interpretation of TVA that does not depend on any specific anatomical localization of TVA computations. However, Bundesen et al. (2005) have suggested a thalamic model of NTVA in which attentional capacity depend on the functional interconnection of thalamic nuclei and visual processing units in the occipital and parietal lobes. In particular, it is suggested that η values from the geniculo-striate pathway is integrated with pertinence values from fronto-parietal cortical systems in a priority map that represent the attentional weights of objects in the visual field. Possibly, the priority map is localized in the pulvinar nucleus in the posterior thalamus. The pulvinar nucleus has been implicated in processes related to visual attention in several studies (Petersen et al., 1987; Saalmann et al., 2012, for a review see Saalmann and Kastner, 2011), and the pulvinar is connected with posterior visual regions via WM tracts that pass through the internal capsule, particularly PLIC and RLIC. In NTVA, it is suggested that the end product of the first wave of selection is the attentional weights that are stored in the priority map. In the second wave of selection, this information is multiplied with β-values in the cortex, the product of which is transmitted to a VSTM map, possibly located in the reticular nucleus in the thalamus. Thus, WM tracts that connect the thalamus with cortical regions should be closely related to performance on TVA-based assessment. Projection fibers were strongly related to performance data. In particular, the IC, SS, and CR ROIs were strongly associated with *t*_0_, and this relation was stronger and more specific as age increased, particularly for the oldest participants (70–80 years) for which *t*_0_ and MD had the strongest effect. However, sample size becomes limited if we focus narrowly on the oldest participants and we have chosen not to emphasize such analyses here. Controlling for age and age^2^ in the regional correlation analysis revealed that only the IC (particularly PLIC) and CR (particularly ACR) remained significant. Furthermore, TVA parameters were associated with fiber tracts that inter-connect frontal, parietal, and occipital cortical regions, including the sagittal stratum and the SLF. Thus, the current data appear to be generally consistent with some of the predictions likely to be made based on NTVA. However, there are a number of qualifications to this interpretation. For example, the associations were not very specific—although some tracts were more strongly associated than others, there was a gradient of correlation strengths, making the associations quantitatively, rather than qualitatively different from each other. Statistical comparison of correlation coefficients could not contribute to specification beyond what is evident in Tables 6, 7. Furthermore, the directionality of connections between brain regions cannot be inferred from DTI images. Finally, there is no way of inferring the sequence of involvement for each region of the brain in correlational studies like this.

### Do the TVA parameters mediate the relationship between DTI indices, processing speed, and fluid intelligence?

To assess the extent to which TVA parameters mediated the relationship between DTI indices, processing speed, and fluid intelligence, we analyzed Baron and Kenny's (1986) four criteria for mediation. Similar to the approach for DTI-mediation of age effects on TVA parameters, we performed the analyses in young and old subsamples. In the young group, FA was correlated with *DSS* and *C*, also after controlling for age and age^2^ (criteria 1 and 2). The *C-DSS* correlation was significant after controlling for age, age^2^, and average FA (criterion 3), and the FA-*DSS* correlation was clearly reduced when *C* was controlled for (criterion 4). Thus, according to these criteria, *C* partly mediated the relationship between FA and *DSS* performance in young participants. This correlation seemed to be primarily driven by the anterior parts of the corpus callosum and corona radiata.

In the old group, MD was correlated with *DSS* and *g*F, also after controlling for age and age^2^ (criteria 1 and 2). The *t*_0_-*g*F correlation, but not the *t*_0_-*DSS* correlation, was significant after controlling for age, age^2^, and average MD (criterion 3), and the MD-*g*F correlation was clearly reduced when *t*_0_ was controlled for (criterion 4). Thus, according to Baron and Kenny's criteria, *g*F were partly mediated by *t*_0_ in participants above the age of 50. The regional source of this correlation was quite distributed and seemed to be strongest in projection fibers and the SLF.

In the present study we had concurrent psychometric, psychophysical (TVA), and DTI data from a relatively large sample of healthy individuals covering the adult age range. This enabled us to make direct comparisons between multicomponent and subcomponent indices of processing speed with regards to correlations with age and with WM structural connectivity. As expected, age effects were larger on the multicomponent measures, than on subcomponents, but association with FA was similar in magnitude, and the relation between FA and *DSS* was partially mediated by *C*. Furthermore, the pattern of association with individual WM tract ROIs was similar for the two measures. This may be taken to indicate that, at least for participants under the age of 50, *DSS* is sensitive to the same neurobiological factors as the computationally more narrowly defined *C* parameter, but that it also captures variance that is unrelated to processing speed *per se*. This can be inferred from the significant correlations with the other TVA parameters, in particular *K* and *t*_0_. It also seems likely that *DSS* captures variance related to computations related to for example memory, executive functions, and motor planning and execution that were not individually estimated in the current study (Ratcliff, 1978).

The latter point is underscored by comparison with the results in the older cohort in which the *DSS* correlations with *K* and *C* are clearly reduced, whereas the correlation with *t*_0_ is essentially unchanged. Also, whereas correlations with WM integrity estimates were similar for *DSS* and *C* for young participants, for old participants it was similar for *DSS* and *t*_0_. However, since *t*_0_ and *DSS* were not significantly correlated when sex, age, age^2^, and average MD were controlled for, we were not able to show that *t*_0_ mediated the correlation between *DSS* and average MD.

Processing speed is a well-known correlate of fluid intelligence (Deary, 2000; Jensen, 2006). In the context of cognitive aging, it has been claimed that decline in higher cognitive functions is largely determined by the efficiency with which simple mental operations can be correctly completed (Salthouse, 1996). However, studies cited in support for this idea has typically employed computationally complex paper-and-pencil tests such as the *DSS*, and as argued above, it is not clear that it is the processing speed aspect of *DSS* that is associated with fluid intelligence. By use of tasks in which performance is unrelated to motor components, one has tried to more unambiguously connect age-related decline in processing speed to age-related decline in fluid intelligence. For example, Ritchie et al. (2014) analyzed longitudinal cognitive aging data from a large sample of healthy individuals in their 70s that had been tested three times with an IT task and other psychometric measures, showed that IT and *g*F was significantly correlated at baseline (*r* = 0.46). Interestingly, when looking at performance changes (i.e., slopes) over a six-year period, the correlation was much stronger (*r* = 0.78), suggesting that there really is a functional connection between age-related declines in perceptual processing speed and fluid intelligence. However, as argued above, and elsewhere (McAvinue et al., 2012; Habekost et al., 2013), IT paradigms cannot distinguish between processing speed (the rate of perceptual processing) and perceptual threshold (the delay of processing onset after stimulus presentation). In light of the results in the present study, an alternative interpretation of the results of Ritchie et al. (2014), and indeed of the processing speed theory of cognitive aging (Salthouse, 1996), is that at least part of the correlation between decline in processing speed and decline in higher order cognition, is due to an age-related elevation in the perceptual threshold. The finding that *t*_0_ partially mediated the correlation between WM diffusivity and *g*F, and that the regional pattern of correlations with WM tract integrity indices seemed to be largely similar for *t*_0_ and *g*F, with relatively stronger correlations for projection fibers, may be taken to support the view that there is a common biological substrate for these measures.

## Conclusion

The findings on the neuroanatomy of TVA are generally consistent with the thalamic model in NTVA, and thus provide convergent evidence. Furthermore, the results may give clues to the source, in neuroanatomical and computational/information-processing terms, of age-related decline in multicomponent measures of processing speed and fluid intelligence. Before the age of 50, age effects on *DSS* may be mediated by changes in FA and *C*. After the age of 50, age effects on *g*F, may be mediated by changes in WM diffusivity, particularly in projection fibers, and *t*_0_. This set of findings is consistent with the notion that the aging brain is characterized by cognitive slowing due to WM tract disconnectivity, as has been argued previously by several investigators (Madden et al., 2012; Penke et al., 2012a,b; Haász et al., 2013). The current study takes this analysis one step further by showing that the association between DTI and processing speed, or between DTI indices and fluid intelligence is partly mediated by more specific computational processes as defined by *C* and *t*_0_, and that these associations differ across the adult age range.

## Limitations

There are several limitations associated with the current study. One limitation is connected to the problem with interpretation of cognition-brain structure correlations. First of all, the data is correlational, so the presence of a significant association does not imply causation. Correlations may be due to other measured or unmeasured variables. Furthermore, it does generally not follow that a lack of correlation means that there is no functional relation between a certain behavioral measure and the anatomical properties of a structure. It has often proved challenging to reveal behavior-brain structure correlations (Van Petten, 2004). Although a specific cognitive function may be dependent on a particular brain substrate, the relation may not emerge in correlation analysis unless the structure is sufficiently different from normal or until a sufficient amount of damage has accumulated (Westlye et al., 2012a). However, structural connectivity is only part of a story where functional or synaptic connectivity may be even more important for certain cognitive processes (Friston, 1998). The importance of WM vs. synaptic connectivity may vary between behavioral measures. For TVA parameters, one could speculate whether *K* and *α* might be more strongly related to synaptic connectivity.

Another limitation is the sample size. Although we are not aware of published studies with larger samples with concurrent TVA-based assessment data and DTI, there are limitations to the ability to split into subgroups for more specific follow-up analyses. Future studies should aim for even larger sample sizes if correlational analyses are to be more decisive. Lastly, acquisition of behavioral data and DTI scanning was done about one year apart. We statistically controlled for differences in the elapsed time between the two types of data sampling, and the effects of this variable did seem to be negligible. However, future studies should aim to do TVA-based assessment and MRI scanning within as short a period of time as possible.

### Conflict of interest statement

The authors declare that the research was conducted in the absence of any commercial or financial relationships that could be construed as a potential conflict of interest.
